# Adverse childhood experiences, loneliness, and doomscrolling on social media newsfeeds among adult men across Generations X, Y, Z

**DOI:** 10.3389/fsoc.2026.1700210

**Published:** 2026-02-27

**Authors:** Sabina Lissitsa, Maya Kagan

**Affiliations:** 1 School of Communication, Ariel University, Ariel, Israel; 2 School of Social Work, Ariel University, Ariel, Israel

**Keywords:** adverse childhood experience, doomscrolling, Generation X, Generation Y, Generation Z, generational cohort theory, loneliness

## Abstract

**Introduction:**

Drawing on compensatory and compulsory internet use theories, media system dependency theory, and generational cohort theory, and informed by scholarship on generationally differentiated masculine norms and gendered socialization, this study examines the role of loneliness in the relationship between adverse child hood experiences (ACEs) and doomscrolling among Israeli men from Generations X (born 1965–1980), Y (born 1981–1996), and Z (born 1997–2006).

**Methods:**

Using a cross sectional research design, data were collected from 570 Hebrew-speaking men using validated self-report measures.

**Results:**

Findings reveal that among Gen X, ACEs are linked to loneliness but not to doomscrolling. For Gen Y, ACEs predict doom scrolling directly, without mediation of loneliness. In contrast, Gen Z shows a distinct mediating role of loneliness in the ACE-doomscrolling link, reflecting compensatory digital coping.

**Discussion:**

This study reveals generational distinctions in both the emotional mechanisms underlying doomscrolling and the broader role of digital media in the lives of men. Interpreted through the lens of generationally molded masculinities, these distinctions help contextualize differences in digital media use across cohorts, with doomscrolling representing one domain in which early adversity may be reflected later in life.

## Introduction

Adverse childhood experiences (ACEs) may arise from many different sources reflecting environmental factors (e.g., living in poverty or unsafe neighborhoods) or relational factors, ranging from emotional, physical, or sexual abuse to disturbed parent–child interactions (e.g., neglect, disengagement from children, as well as hostility and coercion) (Rebbe et al., 2017). While early scholarship on ACEs predominantly addressed their immediate developmental and health-related consequences (Dube et al., 2003; Flaherty et al., 2013; Thompson et al., 2020), a growing body of research has shifted attention to their *enduring* impact on individuals’ social integration, emotional resilience, and relational capacities across the life course (Daníelsdóttir et al., 2022; Lin et al., 2022; Rokach and Clayton, 2023). ACEs can have detrimental and long-lasting effects on health and well-being (Felitti et al., 1998) and are associated with a wide range of negative outcomes later in life, including poor mental (Schilling et al., 2007) and physical health (Monnat and Chandler, 2015), substance abuse (Zhu et al., 2023), interpersonal difficulties (Poole et al., 2018), impaired socio-economic functioning (Currie and Spatz Widom, 2010), chronic loneliness as a stable psychosocial condition (Landry et al., 2022) and even mortality risk (Curtis et al., 2025a).

As the long-term emotional and relational consequences of ACEs become more evident, contemporary scholars have begun to explore how individuals cope with these lingering effects, particularly through digital means (Chaudhary et al., 2024; Hao et al., 2024). Individuals often employ various stimuli, including Internet, to compensate for or escape from life stress (Caplan, 2010; Kardefelt-Winther, 2014). Research has found a correlation between ACEs and problematic Internet (Arslan, 2017; Yates et al., 2012) or mobile use (Li et al., 2020). One specific form of problematic social media engagement that has drawn increasing public and scholarly attention is *doomscrolling* (Cassidy, 2022; Garcia-Navarro, 2020). Doomscrolling refers to the compulsive consumption of negative or distressing news content by continuous scrolling on digital platforms, particularly social media (Sharma et al., 2022). In the current age of digital ubiquity, where access to breaking news stories is free and “on demand” in most online sources, this behavior has become widespread and even normalized (Shabahang et al., 2021). Beyond its psychological correlates, recent work conceptualizes doomscrolling as a pattern of sustained exposure to tragic news characterized by difficulty disengaging from newsfeeds, even when continued consumption is perceived as unhelpful or overwhelming (Shabahang et al., 2023). This perspective highlights disengagement failure as a defining feature of doomscrolling, distinguishing it from general news consumption. Emerging evidence suggests that doomscrolling may carry significant psychological costs, including heightened symptoms of anxiety, depression, and post-traumatic stress (Rodrigues, 2022).

Despite the growing interest in these phenomena, little research has focused specifically on how *men* navigate the long-term consequences of ACEs through digital behaviors such as doomscrolling. This gap is noteworthy given that dominant masculine norms often discourage emotional expression and interpersonal help-seeking (Reidy et al., 2016). Such norms may constrain relational coping pathways, thereby amplifying reliance on internalized and self-directed regulatory processes that operate between early adversity and later behavioral responses (Genuchi et al., 2025). Recent research further suggests that loneliness may heighten attentional sensitivity to threat-related cues (Cacioppo et al., 2016), increasing vigilance toward negative or alarming information. Within this context, doomscrolling may serve as an individually enacted pattern of sustained threat monitoring, particularly when engagement with distressing news content is perceived as informative or preparatory rather than avoidable. Empirical studies have linked doomscrolling to heightened existential concerns and pessimistic worldviews, underscoring its entanglement with threat, trauma, and perceptions of societal instability (Shabahang et al., 2024). Understanding gendered patterns of coping is therefore critical for identifying how psychosocial mechanisms shape men’s engagement with maladaptive forms of media use.

In this vein, evolving definitions of *masculinity*, shaped by changing societal norms, gender roles, and digital technologies (Addis et al., 2016; Gangal et al., 2024; Green and McClelland, 2019) underscore that men’s interpretations of childhood adversity and their coping are socially and culturally situated rather than uniform (Lehrner and Yehuda, 2018; Lurie-Beck, 2007). Prior research indicates that exposure to early-life adversity and its psychological sequelae are embedded within broader life course and socialization contexts, which vary across historical periods and cultural settings (Lissitsa and Kagan, 2024; Garrido, 2020). Existing studies have lacked an integrative approach reflecting the effects of recent global, political, social, and technological changes on these dynamics. Examining these dynamics across three generations: Generation X (born between 1965 and 1980), Millennials (Generation Y, born between 1981 and 1996), and Generation Z (born between 1997 and 2010) can reveal the influence of different social, political, and technological contexts on these relationships.

Although links between ACEs and problematic media use have been observed (Arslan, 2017; Li et al., 2020; Yates et al., 2012), the mechanisms underlying this association remain insufficiently theorized. One plausible pathway is *loneliness* - a lingering sense of social disconnection (Perlman and Peplau, 1998; Pinquart and Sorensen, 2001), that often arises from early relational trauma and functions as an intervening psychosocial mechanism rather than an outcome (Kovacic and Orso, 2024; Lin and Chiao, 2022). Recent mediation-based research supports the conceptualization of loneliness as a mechanism linking ACEs to maladaptive coping and self-regulatory processes (Deng et al., 2024; He and Xiang, 2022; Leng and Dong, 2025; Lin et al., 2022), culminating in a heightened tendency to seek inward-oriented forms of digital engagement (Kovacic and Orso, 2024; Lin and Chiao, 2022), and solitary digital coping strategies such as doomscrolling. This pathway may be particularly salient among men, whose emotional expression is shaped by social expectations pertaining to masculinity (Connell and Messerschmidt, 2005).

The aim of this study is to fill these gaps in the literature by exploring the mediating role of loneliness in the relationship between ACEs and doomscrolling among men from three generational cohorts. By focusing the analysis on the intersection of developmental adversity, digital coping, and shifting masculine norms, this study contributes to a more comprehensive understanding of how sociocultural and temporal contexts shape men’s behavioral and emotional responses. Exploring these dynamics can yield valuable insights for developing more nuanced and effective therapeutic interventions tailored to men navigating these challenges.

## Literature review

### Adverse childhood experiences and doomscrolling

ACEs often reflect not only individual trauma but also deeper structural inequalities that shape family life, emotional development, and access to support (Walsh et al., 2019). These early disruptions, whether resulting from neglect, abuse, or household dysfunction, can leave lasting marks on how individuals cope with distress and engage with the world around them (Rudenstine et al., 2019). Trauma-informed perspectives further suggest that early adversity may foster enduring patterns of repetitive, self-directed coping that resemble addiction-like behaviors, even when no substance use is involved, by shaping how individuals regulate stress and seek relief across the life course (Maté, 2022; Schimmenti et al., 2022). Masculinity norms further complicate this landscape. Cultural expectations that discourage men from expressing vulnerability or seeking emotional support (Connell and Messerschmidt, 2005) often result in the adoption of solitary, indirect coping strategies, like substance use, risk taking behaviors, etc. (Spendelow and Seidler, 2020). These long-term coping patterns, molded by early adversity and gendered expectations, are now manifesting more prominently in digital environments. Increasingly, these coping tendencies take the form of patterns of digital media use, particularly in emotionally driven and compulsive engagement with online content (Chaudhary et al., 2024; Marx et al., 2021).

*Compulsory Internet Use* provides a foundational lens for understanding such digital coping behaviors. Originally conceptualized to describe a persistent, hard-to-control urge to engage with digital content (Meerkerk et al., 2009; van Den Eijnden et al., 2010), this framework emphasizes the habitual and often automatic nature of online activity. Individuals may feel compelled to check news feeds, scroll through social media, or consume content repetitively, even when they recognize the behavior as distressing or counterproductive (Shabahang et al., 2021). In this view, doomscrolling can be seen as a form of digital compulsion, maintained by internal pressure rather than deliberate choice (Kaya and Griffiths, 2024). Such a pattern may be particularly likely to appear among individuals with a history of early adversity, whose disrupted developmental environments fostered a heightened reliance on automatic, habitual behaviors, such as compulsive news consumption, as a means of maintaining a sense of control or vigilance (Sease et al., 2023).

Building on this, *Compensatory Internet Use Theory* (Kardefelt-Winther, 2014) shifts focus to the emotional motivations behind online behavior. It suggests that people often use digital media not simply for entertainment or information, but to escape real life issues, compensate for offline deficiencies such as emotional insecurity, social isolation, or lack of control, particularly when offline resources for coping are lacking. For individuals shaped by early adversity, the internet becomes a space where they can manage distress on their own terms, without the risks that accompany vulnerability in real-life relationships. Doomscrolling may offer an acceptable outlet for emotional distress, by allowing engagement without intimacy and control without disclosure. Repeated consumption of distressing content can offer a paradoxical sense of control or validation in the face of internal chaos and an unpredictable external environment (Lin and Chiao, 2022).

Together, the concepts of compulsory and compensatory internet use provide a nuanced framework for understanding doomscrolling as a behavior shaped by social context, developmental stage, and evolving models of masculinity, where different generations engage in it under the influence of distinct emotional pressures and gendered coping norms.

### Adverse childhood experiences and loneliness

The emotional consequences of ACEs often extend far beyond childhood, shaping how individuals perceive and engage in relationships throughout life (Curtis et al., 2025b). One common outcome of such early relational disruptions is a persistent sense of disconnection from others, which may manifest as loneliness - a subjective experience marked by the perception that one’s social relationships are insufficient in either quality or quantity, and often tied to unmet needs for belonging and attachment (Perlman and Peplau, 1998; Pinquart and Sorensen, 2001).

The link between early adversity and loneliness becomes clearer when considering how children process relational experiences (Lissitsa and Kagan, 2024). Because young children often lack the cognitive maturity to accurately interpret parental behavior, they may internalize mistreatment, leading to distorted self-attributions, diminished self-esteem, and chronic self-blame (Hays-Grudo and Morris, 2020). Such early relational trauma can disrupt the development of secure attachment patterns, erode trust in others, and hinder the acquisition of effective social skills (Hepp et al., 2021). These impairments make it more difficult to form and sustain close relationships later in life, fostering emotional isolation and weakening one’s sense of social connectedness (Tzouvara et al., 2023). Over time, the cumulative burden of social difficulties, chronic stress, and perceived marginalization increases the likelihood of enduring loneliness. Consistent with this trajectory, research shows that ACEs are associated with long-lasting patterns of mistrust, relational withdrawal, and impaired social functioning – factors that significantly elevate the risk of loneliness in adulthood (Colburn et al., 2021; Kisely et al., 2018; Landry et al., 2022).

### Loneliness and doomscrolling

For those who experience persistent loneliness, digital media often serves as a key tool for emotional regulation – a process well explained by Media System Dependency Theory (Ball-Rokeach and DeFleur, 1976). According to this theory, reliance on media intensifies when traditional social supports, such as close relationships or institutional resources, are diminished. In times of uncertainty or emotional strain, media use often extends beyond information-seeking to serve emotional and social functions. When individuals feel lonely or disconnected, they may turn to media to regain a sense of social understanding and orientation - an essential resource for coping with the complexity and ambiguity of contemporary life (Ball-Rokeach et al., 1984; Zhang and Zhong, 2020). To this end, individuals may seek information to make sense of their social environment from a variety of sources, including global news outlets, national television and newspapers, local broadcasts, online platforms, and opinion leaders on social media, often turning to media as a primary coping mechanism that offers structure, reassurance, or a sense of control in an otherwise unpredictable world (Kim and Jung, 2017). However, this reliance can become maladaptive when focused on distressing or emotionally overwhelming content, such as doomscrolling, which tends to surface during times of isolation, insomnia, or prolonged stress (Sharma et al., 2022). While such behavior may offer a temporary sense of engagement or awareness, it frequently reinforces negative affect, increases cognitive load, and deepens feelings of helplessness and despair (George et al., 2024). For individuals already struggling with loneliness, particularly those whose early life experiences, such as ACEs, compromised their ability to form secure relationships, doomscrolling may replicate the pattern of emotional withdrawal and mistrust.

This pattern may be especially pronounced among men, whose socialization often constrains the expression of emotional vulnerability and discourages help-seeking behavior (Reidy et al., 2016). Within dominant norms of masculinity, expressing loneliness or emotional distress is often perceived as a threat to one’s autonomy, strength, or stoic self-image (Lissitsa and Kagan, 2025; Connell, 1995). Consequently, many men may gravitate toward solitary digital behaviors such as doomscrolling as a culturally acceptable means of emotional regulation.

### Generational cohort theory and masculinity

Generational Cohort Theory, originally introduced by Inglehart (1977), organizes populations into generational groups that emerge roughly every two decades—the period it takes for a cohort to grow, come of age, and begin shaping the next generation. The theory posits that individuals who come of age during the same historical period are influenced by similar economic, political, and social forces (Howe and Strauss, 1992), which collectively mold their worldview, social norms, and behavioral tendencies. These formative experiences contribute to the development of a stable generational identity that often endures across the lifespan (Inglehart, 1977; Pekerti and Arli, 2017).

### Generation X

Gen X grew during a period marked by economic volatility, shifting family structures, and significant public health concerns such as the AIDS crisis (Lissitsa, 2025). In this context, children, especially those from divorced families or with two working parents, were often expected to take responsibility and develop independence from an early age. Boys were socialized into dominant models of hegemonic masculinity (Connell, 1995), which prioritized emotional control, resilience, and allegiance to male peers. Pervasive cultural messages such as “Do not cry” and “Be a man” fostered a rigid masculine ideal that discouraged vulnerability and emotional expression (Kaistha and Kumari, 2024). Within this social climate, Gen X men who experienced ACEs may have lacked access to supportive relationships or emotional validation, both within their families and in the broader culture (Hughes and Thomas, 2024). Consequently, they may have developed avoidance-based coping strategies, ignored emotional needs, and struggled to form close interpersonal bonds, heightening their risk of enduring loneliness in adulthood.

As adults, Gen X members experienced the rise of the internet and social media (Çoklar and Tatli, 2021). However, their engagement with these platforms as a means of coping might be minimal due to their delayed exposure to digital technologies (Kagan and Lissitsa, 2023). Their coping preferences are likely to be shaped by earlier patterns of behavior and media use. For Gen X men who experienced ACEs, emotional distress may be more likely to be managed through familiar, analog-era strategies, such as consuming traditional news via television or print, withdrawing into solitary routines, or turning to substance abuse (Choi et al., 2017), rather than engaging in immersive digital behaviors like doomscrolling. As a result, doomscrolling may not resonate with their habitual modes of coping and is thus less relevant in how they navigate the long-term impact of early trauma. Accordingly, we hypothesize:

*H1*: Among Gen X, a positive direct association will be observed between ACEs and loneliness. However, neither ACEs nor loneliness will be directly associated with doomscrolling on social media newsfeeds. Consequently, loneliness will not serve as mediator between ACEs and doomscrolling on social media newsfeeds.

### Generation Y—millennials

Unlike Gen X, Generation Y came of age during a time of relative economic stability and evolving cultural values that increasingly embraced emotional awareness and challenged traditional gender expectations (Twenge, 2014). Boys in this cohort were raised in an environment that encouraged them to break away from conventional masculine roles, fostering more emotionally expressive and relationally open male identities (Green and McClelland, 2019). However, critics like Fox (2016) argue that this shift produced a generation perceived as emotionally fragile and overprotected, potentially undermining their resilience in the face of adversity.

A defining feature of Gen Y’s formative years was the rapid advancement of technology, particularly the widespread emergence of the Internet. Although Gen Y was raised in a more emotionally open environment, their relationship with digital media in general and social media in particular, has taken on a compulsive character (Delińska and Badowska, 2019). Accustomed to multitasking and rapid information retrieval, Gen Y individuals often engage with news content reflexively by consuming updates across multiple platforms as part of a constant, fragmented flow of digital activity (Laor and Galily, 2022). In this context, doomscrolling may reflect a form of compulsory rather than compensatory use, driven more by habitual digital engagement than by efforts to alleviate loneliness. As such, the emotional mechanism of loneliness may be less central to the pathway linking ACEs to doomscrolling among Gen Y men, who may scroll through distressing news content compulsively, regardless of their emotional state or level of social connectedness. Accordingly, we hypothesize:

*H2*: Among Gen Y, loneliness will not mediate the positive direct association between ACEs and doomscrolling on social media newsfeeds, as no direct association will be found between loneliness and either ACEs or doomscrolling on social media newsfeeds in this cohort.

### Generation Z

Gen Z came of age in an era defined by ongoing economic instability, geopolitical unrest, and the omnipresence of social media (Kagan and Lissitsa, 2023; Lissitsa and Kagan, 2024). Growing up amid financial uncertainty, political polarization, and heightened global threats, including terrorism and climate crises, this generation has been shaped by a persistent atmosphere of unpredictability. These conditions have contributed to rising levels of anxiety, a generally cautious outlook on the future, and a diminished sense of trust in institutions and interpersonal relationships (Adamy, 2018).

Gen Z has been identified as the loneliest generation (Cigna’s U.S. Loneliness Index, 2018), with studies suggesting that their heightened exposure to stress-inducing issues such as mass shootings, politics, and current events, often amplified by excessive social media use and the infinite scroll design, contributes to a persistent cycle of doomscrolling and emotional strain (Chisholm and Hartman-Caverly, 2022). Having grown up in a more pluralistic world that supports gender equality and diverse identities, Gen Z has become more empowered to explore and express their individuality (Abbott, 2025; Kosar et al., 2023). The emergence of *fluid masculinity* marked by greater emotional openness and a loosening of traditional gendered expectations (Parks et al., 2021) has facilitated the articulation of vulnerability but has not necessarily translated into access to stable coping resources (Chisholm and Hartman-Caverly, 2022). For those exposed to ACEs, this emotional transparency may deepen the subjective experience of loneliness, making it both more consciously felt and more difficult to contain (*Ibid*). In this context, digital news consumption, particularly via social media, is likely to assume a compensatory function. Gen Z’s engagement with news is more immediate, unfiltered, and algorithmically driven than that of previous generations (Laor and Galily, 2022). Rather than seeking information through structured formats, Gen Z navigates a continuous feed of emotionally intense content, often encountered passively, yet consumed actively. The infinite scroll, push notifications, and short-form content that dominate platforms such as TikTok, Instagram, Telegram and X (formerly Twitter) foster not just sustained exposure, but immersion in distressing global narratives (Kang et al., 2020; Laor and Galily, 2022). In line with Compensatory Internet Use Theory (Kardefelt-Winther, 2014), loneliness may motivate Gen Z individuals to engage with digital media as a means of managing emotional discomfort. For Gen Z, whose media habits are both deeply embedded and emotionally charged, doomscrolling may function as a coping mechanism, offering temporary distraction, validation, or a sense of control in the face of inner turmoil. Within this framework, loneliness emerges as a central mediator, linking early adversity to compensatory patterns of doomscrolling that are not merely habitual, but affectively purposeful.

Accordingly, we hypothesize:

*H3*: Among Gen Z loneliness will mediate the positive direct association between ACEs and doomscrolling on social media newsfeeds. Specifically, a positive direct association will be observed between ACEs and loneliness, which, in turn will be positively and directly associated with doomscrolling on social media newsfeeds.

## Method

### Participants and procedure

The current study was approved by the university’s institutional ethics committee and conducted in accordance with ethical standards for non-clinical research involving human participants. The sample comprised 570 Hebrew-speaking men representing three generational cohorts: Generation X (born 1965–1980), Generation Y (born 1981–1996), and Generation Z (born 1997–2006, aged over 18 at the time of the survey). Participants were recruited through targeted online outreach on social media platforms, primarily Facebook and WhatsApp. Recruitment posts were shared in city- and region-based community groups, as well as in general discussion forums. All recruitment posts were published in publicly accessible groups and no direct invitations were sent to individual members. Data were collected using an anonymous, self-administered online survey administered via the Qualtrics platform. Prior to accessing the questionnaire, participants were presented with an informed consent form detailing the study aims, voluntary nature of participation, and assurances of confidentiality. Only participants who provided informed consent were able to proceed. Eligibility screening was conducted at the beginning of the survey and included confirmation of gender (men) and age (18 to 60). To enhance data quality, the questionnaire included an attention-check item designed to identify inattentive responding; participants who failed this check were excluded from the final analytic sample. Participation was voluntary, and no financial or material compensation was offered.

For sample characteristics and the distribution of research variables, see Table 1.

**Table 1:** Descriptive statistics for the research variables (*n* = 570).

Variables	Gen X (*n* = 132)		Gen Y (*n* = 159)		Gen Z (*n* = 279)	
	*M* (SD)	*N* (%)	*M* (SD)	*N* (%)	*M* (SD)	*N* (%)
Age	53.89 (6.74)		33.91 (4.82)		23.59 (3.23)	
Schooling	15.35 (3.78)		14.60 (2.49)		12.92 (1.52)	
Relationship status						
In a relationship		103 (78)		102 (64.2)		109 (39.1)
Not in a relationship		29 (22)		57 (35.8)		170 (60.9)
Employment						
Employed Not employed		105 (79.5) 27 (20.5)		120 (75.5) 39 (24.5)		208 (74.5) 71 (25.5)
ACEs ^a^	1.04 (1.90)		1.38 (1.99)		1.21 (2.01)	
Loneliness ^b^	4.08 (1.38)		4.60 (1.60)		4.72 (1.66)	
Doomscrolling ^c^	2.00 (0.98)		2.32 (1.16)		2.49 (1.20)	

M = Mean. SD=Standard deviation. ^a^Scores range from 0 to 7, with higher scores indicating exposure to a greater number of categories of childhood abuse and household dysfunction. ^b^Scores range from 3 to 9, with higher scores indicating higher levels of loneliness. ^c^Scores range from 1 to 7, with higher scores indicating higher levels of doomscrolling on social media newsfeeds.

### Measurements

*Adverse childhood experiences* were assessed using the 16-item Adverse Childhood Experiences (ACE; Felitti et al., 1998) questionnaire, designed to assess childhood personal exposure to emotional, physical, and sexual abuse, abuse of one’s mother, substance abuse, mental illness, and incarcerated individuals in the family, among adults, by indicating “yes” or “no” in answer to each question. For example: “While you were growing up, during your first 18 years of life: Did a parent or other adult in the household often: Swear at you, insult you, put you down, or humiliate you, or act in a way that made you afraid that you might be physically hurt?”; “Did you live with anyone who was a problem drinker or alcoholic, or who used street drugs?”

The final index was derived by summing the number of childhood exposure categories endorsed, resulting in scores ranging from 0 to 7. Higher scores reflect exposure to a greater number of categories of childhood abuse and household dysfunction. The internal consistency reliability (Cronbach’s alpha) for this study was 0.796 for Gen X, 0.740 for Gen Y, and 0.778 for Gen Z.

*Self-reported loneliness* was measured using a three-item self-report scale (Hughes et al., 2004) that assessed how often the respondents felt a lack of companionship, left out, and socially isolated. Participants responded using a three-point scale: (1) hardly ever, (2) some of the time, and (3) often. The final loneliness score was obtained by summing responses across the three items, yielding a total score ranging from 3 to 9, where higher scores reflected greater levels of perceived loneliness. The internal consistency reliability (Cronbach’s alpha) for this study was 0.835 for Gen X, 0.796 for Gen Y, and 0.803 for Gen Z.

*Doomscrolling* behavior on social media newsfeeds was assessed using a 15 item Doomscrolling Scale (Sharma et al., 2022). Participants rated each item on a Likert-type scale ranging from 1 (Strongly disagree) to 7 (Strongly agree). For example: “I feel an urge to seek bad news on social media, more and more often”; “I constantly refresh my newsfeeds to see if something bad happened”; “I stay up late at night trying to find more negative news”. The final doomscrolling on social media newsfeeds index was computed by averaging responses across all items, with higher scores indicating greater engagement in doomscrolling behavior. The internal consistency reliability (Cronbach’s alpha) for this study was 0.880 for Gen X, 0.896 for Gen Y, and 0.907 for Gen Z.

Participants` age, schooling, relationship and employment status were also collected as background variables.

### Data analysis

Importantly, although the present study employs a cross-sectional design, the conceptual ordering of the variables is theoretically and temporally grounded. ACEs, by definition, refer to time-bounded events that occurred in childhood and therefore temporally precede both loneliness and doomscrolling on social media newsfeeds, which are assessed in adulthood. Loneliness is conceptualized here as a relatively stable and enduring psychosocial condition that often emerges as a long-term consequence of early relational adversity, rather than as a transient affective state. Doomscrolling on social media newsfeeds, in turn, reflects a contemporary behavioral pattern shaped by ongoing emotional regulation processes. Accordingly, the mediation model is used to examine theoretically ordered associations rather than to infer causal relationships.

**Figure 1:**
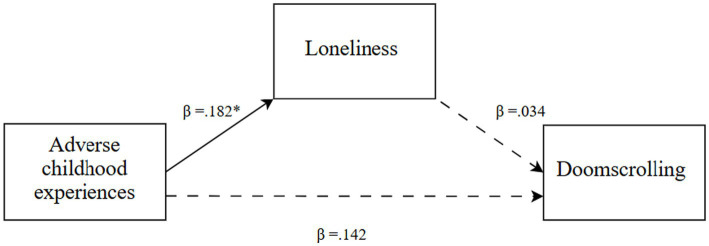
Generation X: a mediation model (Hayes, 2022; Model 4). The solid lines represent significant associations, while the dashed lines represent non-significant associations. ^*^p < 0.05.

**Figure 2:**
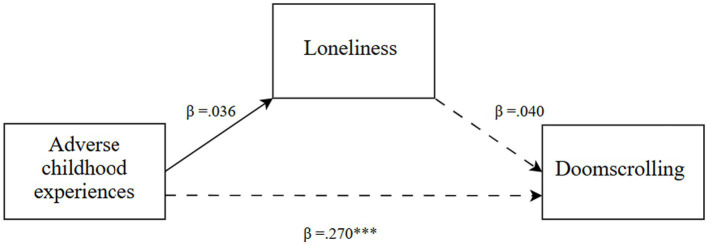
Generation Y: a mediation model (Hayes, 2022; Model 4). The solid lines represent significant associations, while the dashed lines represent non-significant associations. ^***^p < 0.001.

**Figure 3:**
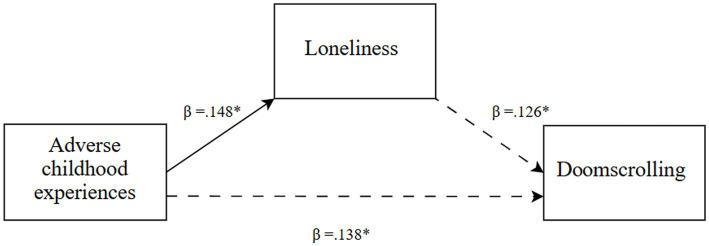
Generation Z: a mediation model (Hayes, 2022; Model 4). Note: The solid lines represent significant associations. ^*^p < 0.05.

Descriptive statistics, including means and standard deviations, were calculated for the research variables. One-way ANOVAs were conducted to examine differences across the three generations in ACEs, loneliness, and doomscrolling on social media newsfeeds. Where the ANOVAs indicated significant overall differences, Tukey’s Honest Significant Difference (HSD) tests were applied to identify specific between-generation contrasts. Also, model 4 PROCESS v4.1 macro for SPSS (Hayes, 2022), was utilized for each generational cohort, with ACEs as the independent variable (X), loneliness (M1) as the mediator, and doomscrolling on social media newsfeeds as the dependent variable (Y). The analysis involved 10,000 bootstrap samples with a 95% confidence interval (Figures 1–3).

## Results

One-way analyses of variance (ANOVA) were conducted to examine generational differences (Gen X, Gen Y, and Gen Z) in adverse childhood experiences, loneliness, and doomscrolling on social media newsfeeds. The analysis revealed no significant generational differences in ACEs [*F*_(2, 567)_ = 1.06, *p* = 0.348]. In contrast, significant generational differences were found for loneliness [*F*_(2, 567)_ = 7.44, *p* < 0.001]. *Post hoc* comparisons using Tukey’s HSD indicated that Gen X reported significantly lower levels of loneliness than Gen Y (mean difference = −0.52, *p* = 0.015) and Gen Z (mean difference = −0.64, *p* < 0.001). No significant difference was found between Gen Y and Gen Z. Significant generational differences were also observed for doomscrolling on social media newsfeeds [*F*_(2, 567)_ = 7.99, *p* < 0.001]. Tukey HSD *post hoc* tests showed that Gen X reported significantly lower levels of doomscrolling than Gen Y (mean difference = −0.32, *p* = 0.046) and Gen Z (mean difference = −0.48, *p* < 0.001). The difference between Gen Y and Gen Z was not statistically significant (for means and standard deviations see Table 1).

### Hypotheses testing

#### Gen X

In line with the research hypothesis (*H1*), ACEs were directly and positively associated with loneliness (*β* = 0.182, t = 2.11, *p* = 0.037). However, the indirect association between ACEs and doomscrolling on social media via loneliness was not significant (*β* = 0.006, Boot SE = 0.020, Boot LLCI = −034, Boot ULCI = 0.050), as neither the direct association between ACEs and doomscrolling nor the direct association between loneliness and doomscrolling was significant (*p* > 0.05).

#### Gen Y

The research hypothesis (*H2*) was fully confirmed. ACEs were directly and positively associated with doomscrolling on social media newsfeeds (*β* = 0.270, *t* = 3.51, *p* < 0.001), whereas the indirect association via loneliness was not significant (*β* = 0.001, Boot SE = 0.008, Boot LLCI = −0.015, Boot ULCI = 0.020), as loneliness was not directly associated with either ACEs or doomscrolling (*p* > 0.05).

#### Gen Z

In line with the research hypothesis *(H3)*, ACEs were directly and positively associated with doomscrolling on social media newsfeeds (*β* = 0.138, *t* = 2.31, *p* = 0.022), with a significant indirect association via loneliness (*β* = 0.019, Boot SE = 0.012, Boot LLCI = 0.001, Boot ULCI = 0.047). Specifically, ACEs were directly and positively associated with loneliness (*β* = 0.148, *t* = 2.49, *p* = 0.013), which, in turn, was directly and positively associated with doomscrolling (*β* = 0.126, *t* = 2.11, *p* = 0.036).

For regression coefficients see Table 2.

**Table 2:** Regression coefficients and standard errors in the mediation model (*n* = 570).

Variables	Independent variables		Dependent variables				
Generation		*B*	SE	*β*	*T*	95%CI	*R* ^2^
Gen X		Loneliness (M1)					
	ACEs	0.132	0.063	0.182	2.11^*^	[0.008, 0.256]	0.033^*^
	Doomscrolling on social media newsfeeds (Y)						
	ACEs	0.073	0.045	0.142	1.61	[−0.017, 0.163]	0.023
	Loneliness	0.024	0.062	0.034	0.38	[−0.099, 0.148]	
Gen Y		Loneliness (M1)					
	ACEs	0.029	0.064	0.036	0.45	[−0.098, 0.155]	0.001
	Doomscrolling on social media newsfeeds (Y)						
	ACEs	0.157	0.045	0.270	3.51^***^	[0.069, 0.246]	0.076^**^
	Loneliness	0.029	0.056	0.040	0.52	[−0.081, 0.139]	
Gen Z		Loneliness (M1)					
	ACEs	0.122	0.049	0.148	2.49^*^	[0.025, 0.218]	0.022^*^
	Doomscrolling on social media newsfeeds (Y)						
	ACEs	0.082	0.036	0.138	2.31^*^	[0.012, 0.152]	0.040^**^
	Loneliness	0.091	0.043	0.126	2.11^*^	[0.006, 0.176]	

^*^p < 0.05; ^**^p < 0.01; ^***^p < 0.001.

## Discussion

This study examined the mediating role of loneliness in the relationship between ACEs and doomscrolling among Israeli men from three generational cohorts: Gen X, Gen Y and Gen Z. For *Gen X*, the association between ACEs and loneliness, without a corresponding link to doomscrolling, suggests that emotional distress persists into midlife but is not typically expressed through digital coping. From a theoretical perspective, masculine ideals during their formative years emphasized stoicism, self-reliance, and emotional restraint (Connell, 1995). Men who went through ACEs often endured their pain in silence, as prevailing norms discouraged disclosure, vulnerability, or emotional validation (Connell and Messerschmidt, 2005). Although masculinity norms were not directly measured in the present study, the pattern observed suggests that, in adulthood, while Gen X men report elevated loneliness linked to earlier ACEs, this distress does not translate into compulsive or compensatory doomscrolling behavior. This absence may reflect a continued adherence to analog-era coping mechanisms and a reluctance to seek comfort in digital spaces (Çoklar and Tatli, 2021). At their current life stage, these men may remain emotionally withdrawn, relying instead on solitary routines or internalized coping strategies. In this context, doomscrolling simply does not align with their established emotional repertoire.

In contrast, the results for *Gen Y* indicate that ACEs are directly linked to doomscrolling, though not through loneliness. Gen Y emerged at a transitional moment in the cultural evolution of masculinity (Green and McClelland, 2019). Raised in a more emotionally expressive environment than Gen X (Twenge, 2014), but still bearing the residual weight of traditional masculine expectations, Millennial men are gripped in a tension between openness and self-discipline. Their coming of age coincided with the digital revolution, when information became ubiquitous, connectivity constant, and multitasking habitual (Çoklar and Tatli, 2021). For this cohort, doomscrolling appears to serve a compulsive function (Kaya and Griffiths, 2024), detached from emotional needs such as loneliness. Rather than acting as a response to distress, it becomes part of a broader pattern of habitual media use. While ACEs remain significant, the results suggest that the pathway linking early adversity to doomscrolling in this cohort is not primarily affective. For Gen Y, digital media represents routine rather than refuge. This reinforces the impulse to remain “in control” by maintaining an endless stream of activity but without directly confronting emotional discomfort. Compulsive scrolling becomes a behavioral echo of masculine restraint. It is active, persistent, and emotionally detached.

Among *Gen Z,* findings show that both ACEs and loneliness predict doomscrolling, with loneliness mediating the relationship between early adversity and the dependent variable. In contrast to the two earlier generations, Gen Z reflects a masculinity that is more fluid, emotionally expressive, and attuned to personal vulnerability (Lissitsa & Kagan, 2025). Gen Z men grew up in a sociopolitical climate characterized by instability, hyperconnectivity, and identity pluralism (Adamy, 2018). For this generation, doomscrolling may serve a compensatory function (Kardefelt-Winther, 2014). It is a digitally mediated attempt to self-soothe, process distress, or reclaim a sense of control. Compulsive engagement with distressing news content becomes a way to manage emotional turmoil in private, without risking social exposure.

The role of Media System Dependency Theory (Ball-Rokeach and DeFleur, 1976) is particularly salient for Gen Z. When traditional sources of support (e.g., secure attachments, institutional trust, or face-to-face interactions) are weak or absent, especially in the wake of early adversity, media becomes a primary resource for meaning-making and emotional regulation. Consistent with this framework, Gen Z’s doomscrolling reflects a search for coherence in a world they experience as fragmented, uncertain, and often threatening (Adamy, 2018). However, this behavior often produces the opposite effect: by gravitating toward negative news, individuals may enter self-reinforcing cycles of emotional harm, intensifying psychological distress rather than achieving clarity, safety, or relief from loneliness, particularly when engagement with negative news lacks intentional regulation or mindful disengagement (Shabahang and Weber, 2025). Among Gen X and Y, media does not fulfill the same psychological or relational role. For these cohorts, traditional or analog forms of meaning-making still persist, suggesting that media dependency processes are not uniform across cohorts.

Taken together, the findings underscore the importance of understanding doomscrolling not as an online behavior leading to negative consequences (Musiał, 2025; Sharma et al., 2022), but as one that is deeply embedded in socialization processes, masculine identity development, and media ecologies. While Gen Y men may engage in doomscrolling primarily as a function of habitual use and information overload, a pattern that can be interpreted as aligning with masculine scripts that discourage emotional engagement, Gen Z men’s doomscrolling may reflect an attempt to feel, process, and contain emotional distress. And Gen X, despite exposure to negative experiences and elevated loneliness, may largely remain outside the digital loop, a pattern that can be interpreted as consistent with masculine norms that discourage emotional disclosure and the use of digital spaces for compensation.

The findings reveal generational distinctions in both the emotional mechanisms underlying doomscrolling and the broader role digital media plays in the lives of men shaped by differing masculine norms and socialization patterns. While masculinity and socialization provide a useful interpretive framework for contextualizing these differences, future research incorporating direct measures of masculinity norms is needed to empirically test these explanations.

### Theoretical contributions

This study offers several theoretical contributions to the intersection of trauma research, digital media studies, and masculinities across generations. First, it extends Compensatory Internet Use Theory by suggesting that digital coping strategies, such as doomscrolling, are not only shaped by individual psychological states but also by generational identity and gendered socialization. While existing literature has framed doomscrolling largely as a response to affective distress (Saindon, 2021), the present findings indicate that its function may vary across generations, potentially reflecting differences in socio-technological developmental contexts, habitual media engagement, and gendered norms of emotional regulation. This generational lens adds nuance to existing interpretations of why individuals engage in maladaptive digital behaviors, and for whom such behaviors serve an emotionally meaningful purpose.

Second, the study refines the application of Media System Dependency Theory by situating it within a developmental and cohort-specific framework. Whereas previous applications have emphasized media dependency during moments of societal crisis or institutional distrust (Khalifa and Khalifa, 2020), the current findings suggest that the theory may be particularly applicable to Gen Z, whose lived experience is marked by both persistent global instability and emotional openness. For this cohort, digital news media may function as a salient resource for emotional regulation and social orientation.

Third, the study advances masculinity theory by offering an exploratory examination of how different models of masculinity may intersect with ACEs-related processes and digital behavior across the life course. The findings are interpreted within a broader socio-cultural context in which gendered norms of masculinity function as a generational backdrop for understanding observed patterns across cohorts. By integrating masculinity theory with generational cohort theory and digital behavior, the study proposes a more temporally sensitive and intersectional framework for understanding male coping in the context of early adversity.

Finally, this research contributes to generational theory by illustrating how macro-level historical forces, such as technological change, shifting gender norms, and socio-political upheavals, may become embedded in individual patterns of digital media use and emotional regulation. These findings underscore the importance of considering digital coping strategies as historically and socially situated, rather than solely individual or pathological responses.

### Practical implications

The results of this study point to the need for differentiated approaches to prevention and intervention, as well as for broader policy frameworks that address the emotional, technological, and generational contexts shaping men’s problematic social media use.

For Gen X, intervention strategies should prioritize emotional literacy and help normalize vulnerability and help-seeking among men socialized under traditional masculinity norms. Community-based mental health programs that facilitate offline connection, such as peer support groups, guided workshops, or local mentorship initiatives, may be particularly effective. Messaging should be delivered through traditional media channels, including television, radio, and print, which are more likely to reach this cohort. At the policy level, integrating trauma-informed practices into national healthcare protocols and funding midlife mental health services can support early detection and intervention among men who may otherwise remain underserved.

In the case of Gen Y, interventions should focus on managing compulsive digital behavior by fostering healthier digital routines and awareness of screen-time overload. Programs can be embedded in organizational wellness initiatives, educational institutions, or digital platforms, emphasizing digital hygiene, mindfulness, and intentional media consumption including strategies that promote more reflective and harmonious engagement with negative news content rather than continuous exposure (Shabahang and Weber, 2025). Policymakers should promote national digital wellness initiatives and encourage employers to adopt evidence-based programs in media literacy and stress management, particularly targeting Gen Y men, who are frequently immersed in high levels of digital exposure.

For Gen Z, practical efforts must recognize the compensatory function of digital media and respond to emotional needs exacerbated by early adversity. Interventions should focus on trauma-informed mental health education, emotional regulation skills, and the development of safe digital and physical spaces where young men can express vulnerability without stigma. Collaborations with content creators and social media influencers should be leveraged to deliver mental health messaging in a tone and format aligned with Gen Z’s digital culture. On the policy level, investments in school-based and online mental health services should be prioritized, including the development of AI-driven tools that identify patterns of distress-related doomscrolling and provide in-app support. National campaigns should explicitly address male loneliness and emotional distress, redefining help-seeking as compatible with emerging, more fluid masculinities.

### Study limitations and recommendations for further research

This study is subject to several limitations that should be acknowledged. First, the use of convenience sampling restricts the representativeness of the sample and limits the generalizability of the findings. Recruiting participants through online platforms may have inadvertently skewed the sample toward individuals with higher digital literacy, greater interest in the subject matter, or a higher degree of comfort discussing psychological and gender-related issues in an anonymous format. As a result, the perspectives captured here may not reflect those of the broader population of Israeli men.

Second, the study’s reliance on a cross-sectional and anonymous design introduces methodological constraints. The inability to collect data across multiple time points or sources (Podsakoff and Organ, 1986) limits the capacity to draw conclusions about the causal direction of the observed relationships, in particular the proposed mediating role of loneliness in the link between ACE and doomscrolling. Although the model is grounded in established theory and prior empirical findings, longitudinal research is needed to clarify the temporal ordering of these variables and to evaluate alternative explanatory models.

The scope of the study was also limited by the number of psychological variables included. Incorporating additional constructs, such as self-esteem, emotional regulation, resilience, coping styles, and perceived social support, would allow for a more comprehensive analysis of the psychological mechanisms at play.

Finally, future research would benefit from employing mixed-method designs. Integrating quantitative data with qualitative narratives could provide deeper insights into how men across generations interpret and manage the long-term consequences of ACEs, especially in the context of shifting gender norms and evolving expressions of masculinity in the digital age.

## Data Availability

The raw data supporting the conclusions of this article will be made available by the authors, without undue reservation.
